# Wet depositions of cations in forests across NADP, EMEP, and EANET monitoring networks over the last two decades

**DOI:** 10.1007/s11356-022-24129-8

**Published:** 2022-11-12

**Authors:** Chung-Te Chang, Ci-Jian Yang, Jr-Chuan Huang

**Affiliations:** 1 Taiwan International Graduate Program (TIGP) – Ph.D. Program on Biodiversity, Tunghai University, Taichung, 407224 Taiwan; 2 Department of Life Science, Tunghai University, Taichung, 407224 Taiwan; 3 German Research Centre for Geosciences (GFZ), 14473 Potsdam, Germany; 4 Department of Geography, National Taiwan University, Taipei, 10617 Taiwan

**Keywords:** Atmospheric deposition, Cations, Nutrient cycling, Forest ecosystem, Neutralization factor (NF)

## Abstract

Studies focused on emissions and acid deposition of sulfur (S) and nitrogen (N) and the consequent precipitation acidity have a long history. However, atmospheric depositions of cations play a critical role in buffering precipitation acidity, and providing cationic nutrients for vegetation growth lacks sufficient studies equally. The spatiotemporal patterns of cation depositions and their neutralization potential across broad scales remain unclear. Through synthesizing the long-term data in forest sites (*n* = 128) derived from three monitoring networks (NADP in Northern America, EMEP in Europe, and EANET in East Asia) on wet deposition of cations (Na^+^, NH_4_-N, K^+^, Mg^2+^, and Ca^2+^), this study assesses the temporal changes and spatial patterns of cation depositions and their neutralization potential over the last two decades. The results showed that the depositions of cationic nutrients were considerably higher in EANET compared to NADP and EMEP. The depositions of sea salt-associated sodium exhibited a significant transition from marine (> 15 kg ha^−1^ year^−1^) to inland (< 3.0 kg ha^−1^ year^−1^) forest sites attributable to the precipitation quantity and influences of sea spray. The higher emissions of NH_3_ and particulate matter in East Asia explained the higher cation depositions in EANET than NADP and EMEP. The annual trends of cations revealed that only 20–30% of the forest sites showed significant changing trends and the sites widely spread across the three networks. Possibly, base cation (BC) deposition has reached a low and stable condition in NADP and EMEP, while it has high spatial heterogeneity in the temporal change in EANET. The difference in BC deposition among the three networks reflects their distinct development of economy. Our synthesis indicates that the annual trends of neutralization factor (NF) in NADP can be explained by the declining of acid potential (AP), not by neutralization potential (NP) as BC deposition has been stably low over the past two decades. Whereas, the concurrent decreases of AP and NP in EMEP or plateau period of both AP and NP in EANET have come to a standstill of acid neutralizing capacity.

**Supplementary Information:**

The online version contains supplementary material available at 10.1007/s11356-022-24129-8.

## Introduction

Base cations (BCs, i.e., calcium [Ca^2+^], magnesium [Mg^2+^], sodium [Na^+^], and potassium [K^+^]) not only support the necessary elements for plant growth, but also decrease the acidity of precipitation through reacting with sulfuric and nitric acid (White and Broadley 2003; Du et al. 2018; Zhang et al. 2020). Although ammonium (NH_4_^+^) is recognized to promote soil acidification by nitrification (Van Breemen et al. 1982), evidences showed that it was also important to neutralizing capacity in precipitation (Rodhe et al. 2002; Huang et al. 2008; Kopáček et al. 2016). Natural sources of cations include sea salts, wind erosion of arid soils, or forest fires, while anthropogenic emissions include industrial productions, agricultural activities, traffic emissions, and unpaved roads (Draaijers et al. 1997; Vet et al. 2014). Adding BCs into the soil will alleviate soil acidification by replenishing BC pool (Larssen and Carmichael 2000; Fenn et al. 2015). Studies indicated that the sharp decreases of BCs in precipitation had negated the positive effects of reduced sulfur (S) and nitrogen (N) depositions which consequently had postponed the pH increase in precipitation in many regions in Europe and North America (Hedin et al. 1994; Kopáček et al. 2016). In contrast, the high precipitation pH in regions with high sulfate (SO_4_^2−^) and nitrate (NO_3_^−^) depositions such as East Asia was attributed to high contribution of ammonia (NH_3_) from agriculture and BCs from natural (soil dust) and industrial (dust emission from coal combustion) sources (Vet et al. 2014).

A recent global synthesis indicates that the decreasing precipitation acidity (increasing precipitation pH) was related to descending sulfur and nitrogen deposition in forests in North America and Europe resulting from the long-term implementation of emission control policies, but no clear trends were found in East Asia (Chang et al. 2022). Previous findings suggested that in regions not significantly influenced by marine or dust aerosols, the emissions of BCs and sulfur dioxides (SO_2_) were closely related. Thus, precipitation acidity may remain relatively stable because depositions of BCs will decrease with stringent S and N emission control policies and lessened anthropogenic activities such as construction and unpaved road improvement of exposed background environment (Fang et al. 2013; Kopáček et al. 2016). The spatiotemporal patterns of BCs in precipitation are prominently involved in acidification, but are rarely examined in continental or global scale compared to SO_4_^2−^ and NO_3_^−^ depositions. Given the role of BCs on buffering acid deposition and in nutrient cycling, broad-scale analysis is critical for modeling ecosystem response to global atmospheric depositions.

**Fig. 1:**
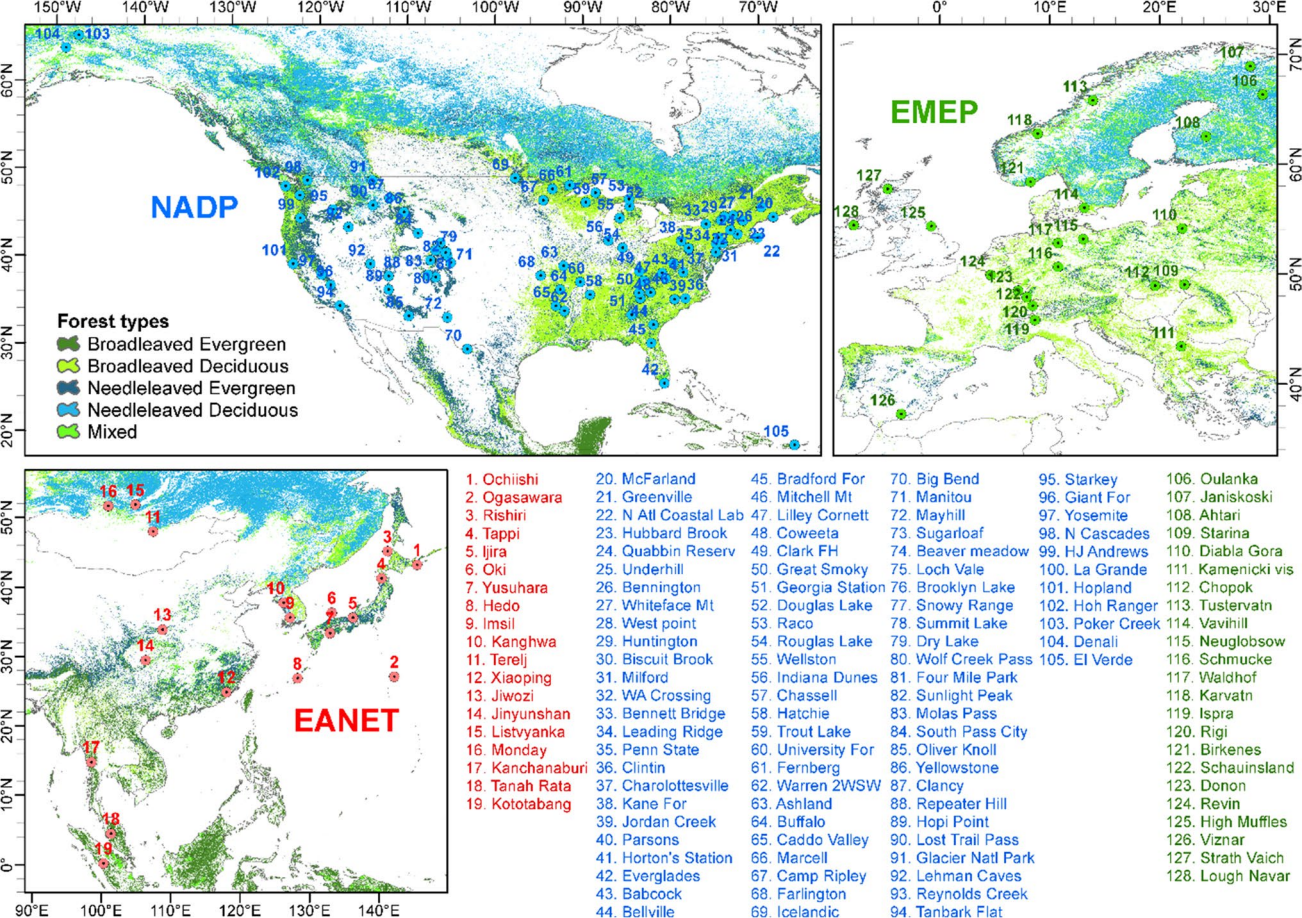
The selected forest sites used of this study from EANET (*n* = 19), NADP (*n* = 86), and EMEP (*n* = 23) acid deposition monitoring networks. (Source of forest types in background: ESA GlobCover 2009 Project: http://due.esrin.esa.int/page_globcover.php, Bicheron et al. 2011)

Forests account for a major cover (> 30%) of global terrestrial ecosystems and play a very crucial role in provision of essential ecosystem services such as conservation of soil and water, mitigation of regional climate, and foundation of diverse habitats (Miura et al. 2015; Sannier et al. 2016). In addition to providing nutrients, the depositions of BCs are particularly important in forests with high acid deposition or low BC pool of soil system for neutralizing capacity. Long-term cross-region continuous monitoring of BCs is required to realize their evolution over time and broad scale, and to explore the role of natural and anthropogenic processes on current depositions and trends. In this study, we synthesized the concurrent observations of BC depositions in 128 forest sites, sites located within national parks, national wildlife refuges, or experimental forests from three continental-scale monitoring networks including National Atmospheric Deposition Program (NADP; *n* = 86) in North America, European Monitoring and Evaluation Programme (EMEP; *n* = 23) in Europe, and the Acid Deposition Monitoring Network in East Asia (EANET; *n* = 19; Fig. 1) over the last two decades. The objectives of this study are to (1) examine the long-term trends of depositions of base cations (BCs) across three networks from 1999 to 2018 and (2) evaluate the role of BCs in neutralizing major acid depositions.

## Materials and methods

### Data acquirement of precipitation chemistry

Annual data of rainfall, pH, concentration, and deposition of SO_4_-S (sulfate sulfur), NO_3_-N (nitrate nitrogen), Ca^2+^, Mg^2+^, Na^+^, K^+^, and NH_4_-N (ammonium nitrogen) were obtained from NADP (1999–2018; https://nadp.slh.wisc.edu/networks/national-trends-network/), EMEP (1999–2015; https://ebas.nilu.no/), and EANET (2001–2018; https://www.eanet.asia/). The datasets of precipitation chemistry were based on weekly collections using wet-only sampling in most sites. The data have been integrated to an annual basis after data processing with standard procedures including ion balance (in μeq L^−1^; i.e., [total cation charge – total anion charge] / [total cation charge + total anion charge] × 100%), and collection efficiency check of annual precipitation > 70% as suggested by the World Meteorological Organization (WMO; Lamb and Bowersox 2000; Allan 2004; Chang et al. 2017). The detail of acceptance ranges of ion balance can be found in the manuals and quality assurance for chemical analysis of each monitoring network (Allan 2004; EMEP 2016; Sakamoto et al. 2016). To examine if there is a consistence between emission and deposition at the global scale, we obtained the emission data from Emissions Database for Global Atmospheric Research (EDGAR v6.1; https://edgar.jrc.ec.europa.eu/dataset_ap61; Crippa et al. 2019, 2020).

Because the depositions of NH_4_-N and cations were largely influenced by NH_3_, PM_10_, and PM_2.5_ (Zhang et al. 2020). The annual time-series of NH_3_, PM_10_, and PM_2.5_ substances and annual grid maps between 1999 and 2018 were acquired from EDGAR v6.1.

### Data calculation

The long-term annual volume-weighted mean (VWM) concentrations and precipitation pH were calculated based on annual rainfall depth and annual mean ionic concentration (Conradie et al. 2016).

The non-sea salt (nss) fractions of Ca^2+^ (nssCa^2+^), Mg^2+^ (nssMg^2+^), and K^+^ (nssK^+^) were processed using Na ratio correction for further analysis and presentation, which excluded the contribution from oceanic source (Jacob et al. 1985; Drever 1988). The ratios of SO_4_^2−^, Ca^2+^, Mg^2+^, and K^+^ to Na^+^ were 0.12, 0.043, 0.023, and 0.021, respectively, in sea salt (Keene et al. 1986; Avila 1996). Studies suggested that the sulfuric and nitric acids together could be defined as acidic potential (AP), whereas total cation concentration of Ca^2+^, Mg^2+^, K^+^, and NH_4_-N represents the major neutralization potential (NP) of precipitation. Based on NP and AP, the neutralization factor (NF = NP / AP) can be derived to evaluate the neutralization capacity of precipitation (Huang et al. 2008; Laouali et al. 2012; Conradie et al. 2016; Adhikari et al. 2021), with values higher than unity suggesting relative high acid neutralization capacity. The precipitation pH is also associated with the difference between NP and AP (Chang et al. 2017). We also consider the non-sea salt contribution (crustal origins and anthropogenic activities) of NP on AP; therefore, the calculation of NF can be expressed as follows:

1 $$\mathrm{NF}=\frac{[{\mathrm{nssCa}}^{2+}+\mathrm{ nss}{\mathrm{Mg}}^{2+}+\mathrm{ nss}{\mathrm{K}}^{+}+ {\mathrm{NH}}_{4}-\mathrm{N}]}{[{\mathrm{nssSO}}_{4}-\mathrm{S }+ {\mathrm{NO}}_{3}-\mathrm{N}]}$$

where the [nssCa^2+^  + nssMg^2+^  + nssK^+^  + NH_4_-N] and [nssSO_4_-S + NO_3_-N] stand for the equivalent concentrations (μeq L^−1^) (Kumar et al. 2002; Keresztesi et al. 2019).

### Statistical analysis

The quantitative trends (annual change rate) of various atmospheric depositions and NF over time were obtained from slopes of the linear square regression models. The statistical significance was determined by Mann–Kendall (MK) test for inter-annual trends as suggested by WMO and has been widely applied in hydro-climatic and environmental studies (Antonopoulos et al. 2001; del Río et al. 2007; Ali et al. 2019). Data normal distribution is not necessary for MK test, and this method is less sensitive to outliers (Tao et al. 2011; Sonali and Kumar 2013).

## Results and discussion

### Spatial patterns of precipitation and depositions of cations

**Fig. 2:**
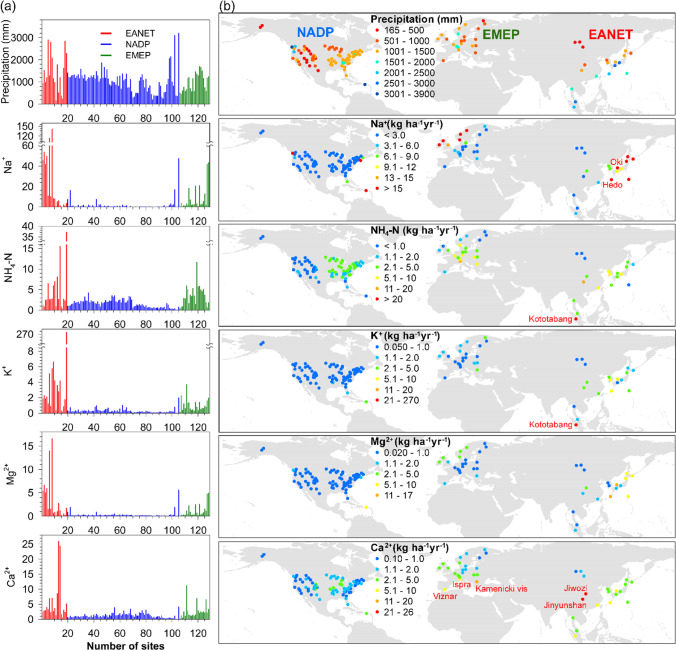
The mean annual precipitation (mm year^−1^) and average of annual depositions (kg ha^−1^ year^−1^) of Na^+^, NH_4_-N, K^+^, Mg^2+^, and Ca^2+^ (**a**), and their spatial patterns (**b**) from top to bottom panels

The ions associated with sea salt, i.e., Na^+^, K^+^, and Mg^2+^, show a clear decreasing pattern from ocean sites to inland sites which can be attributed not only to the influence of sea salt spray but also the gradual decreasing precipitation from the coast to the inland sites (Fig. 2; Fig. S1). For example, the windward sites in temperate altitudes such as northwest America and islands in subtropical/tropical Southeast Asia received rainfall higher than 1500 mm year^−1^, whereas the inland locations in west America, central and east Europe, and central and north Asia have annual rainfall below 500 mm (Fig. 2). The sea salt-associated ions Na^+^ (> 15 kg ha^−1^ year^−1^), K^+^ (> 2.0 kg ha^−1^ year^−1^), and Mg^2+^ (> 2.0 kg ha^−1^ year^−1^) were much higher in the coastal and islands (Figs. 1 and 2). The Oki (site 6) and Hedo islands (site 8) in Japan are two sites with the highest Na^+^ (> 114 kg ha^−1^ year^−1^), K^+^ (> 5.0 kg ha^−1^ year^−1^), and Mg^2+^ (> 14 kg ha^−1^ year^−1^) depositions ascribed to the prevailing monsoon climate with higher precipitation (approximate 3000 mm year^−1^) or were clearly influenced by sea spray and significant contribution by typhoon periods when the air masses originated from pristine ocean (Fujita et al. 2000; Sakihama et al. 2008). By contrast, most inland sites in the three networks show lower depositions of Na^+^ (< 3.0 kg ha^−1^ year^−1^), K^+^ (< 1.0 kg ha^−1^ year^−1^), and Mg^2+^ (< 2.0 kg ha^−1^ year^−1^), reflecting their lower ionic concentrations and precipitation quantity (Fig. 2; Fig. S2).

For NH_4_-N, 60 sites (47%) have annual deposition > 2.0 kg ha^−1^ year^−1^, mainly spread from east to central USA, central to east Europe, and East Asia. These regions are near to arable regions, with high agricultural activities and livestock breeding (Fig. 2; Keresztesi et al. 2019, 2020; Wang et al. 2019). Whereas, West USA, North Europe, and North Asia (68 sites), which are relatively unaffected by anthropogenic activities, had lower NH_4_-N deposition (< 2.0 kg ha^−1^ year^−1^; Fig. 2). Among all locations, Kototabang, Sumatra in Indonesia (site 19) has the highest depositions of NH_4_-N (37 kg ha^−1^ year^−1^) and K^+^ (274 kg ha^−1^ year^−1^) (Fig. 2), with annual deposition as high as 666 and 4830 kg ha^−1^ year^−1^ for NH_4_-N and K^+^, respectively, in 2015 due to forest fires. During September and October 2015, southern Sumatra and Kalimantan experienced the worst fires over the past two decades (Chang et al. 2022), resulting from forest clearing for agriculture use in combination with a prolonged drought due to a strong El Niño (Huijnen et al. 2016; Tacconi 2016). The 2015 fire episodes released large amounts of particulate matter and carbon and contributed to the high depositions of SO_4_-S, NO_3_-N, NH_4_-N, and K^+^ (Fujii et al. 2019; Kiely et al. 2019). The widespread slash-and-burn agriculture in Southeast Asia and the projected increases of El Niño drought events in a warming climate have profound effects on fire frequency and intensity and consequent nutrient cycling (Duncan et al. 2003; Yin 2020).

The Ca^2+^ deposition shows a clear spatial pattern within the NADP network although the level of deposition is low and stable compared to the other two networks. Based on the synthesis of 334 sampling sites across the USA, Keresztesi et al. (2020) reported that Ca^2+^ deposition was relatively high in Western USA such as Colorado, Utah, and Wyoming, mainly contributed by dusts. The high Ca^2+^ deposition in central and eastern USA is attributable to power plant and cement production (Brahney et al. 2013). In addition, the higher deposition of Ca^2+^ might be arisen from anthropogenic emissions including industry, traffic on unpaved roads, or transportation from deserts particularly. Two-decadal average of Ca^2+^ deposition in NADP and EMEP is lower than 5.0 kg ha^−1^ year^−1^, except for three sites, Kamenicki vis (site 111), Ispra (site 119), and Viznar (site 126), near the Mediterranean which have average deposition of 6.6–11.2 kg ha^−1^ year^−1^ (Fig. 2). The regions surrounded by Mediterranean were significantly influenced by dust transportation from Sahara Desert that provides up to one-third of annual Ca^2+^ input, especially during the spring–summer period (Lequy et al. 2012). The Ca-rich dust not only reduces precipitation acidity but also is an important source of Ca^2+^ to many forest ecosystems (Alastuey et al. 1999; Rogora et al. 2004; Sverdrup et al. 2006). The average wet deposition of Ca^2+^ in EANET forests (6 kg ha^−1^ year^−1^) is 2.6 to 4.7 times of the averages in EMEP (2.60 kg ha^−1^ year^−1^) and NADP (1.27 kg ha^−1^ year^−1^) forests (Fig. 2). The two sites with the highest Ca^2+^ deposition in EANET (> 24 kg ha^−1^ year^−1^) are Jiwozi (site 13) and Jinyunshan (site 14) in central China (Figs. 1 and 2), owing to wind-brown dust from arid and semi-arid desert in northwest China and anthropogenic emissions, such as cement production, wind erosion of arable land, traffic on unpaved roads, and fossil fuel combustion (Zhang et al. 2012; Duan et al. 2016; Du et al. 2018). In eastern and southern China, the rapid urbanization and industrialization had significantly contributed to the deposition of cations (Li et al., 2016a). However, there is a decreasing trend of dust storms in northern China over the past decades due to increasing vegetation cover and reducing wind speed (Wang et al. 2017). Recent intensification of droughts caused by higher temperature has caused a rebound of dust emission which might deteriorate the stability of transition zones between semi-arid and arid regions, and human activities will exacerbate dust storm susceptibility and consequent nutrient cation depositions to ecosystems (Song et al. 2016; Al-Najjar et al. 2020; Liu et al. 2020; Yu and Zai 2020; Gross et al. 2021). In addition, air pollution and particulate matter associated with dust storms are very detrimental to human health regardless of their origins and deserve further attention (Hoek et al. 2013; Yang et al. 2013; Zhou et al. 2021; Heft-Neal et al. 2022).

### Temporal trends of cations across three networks

**Fig. 3:**
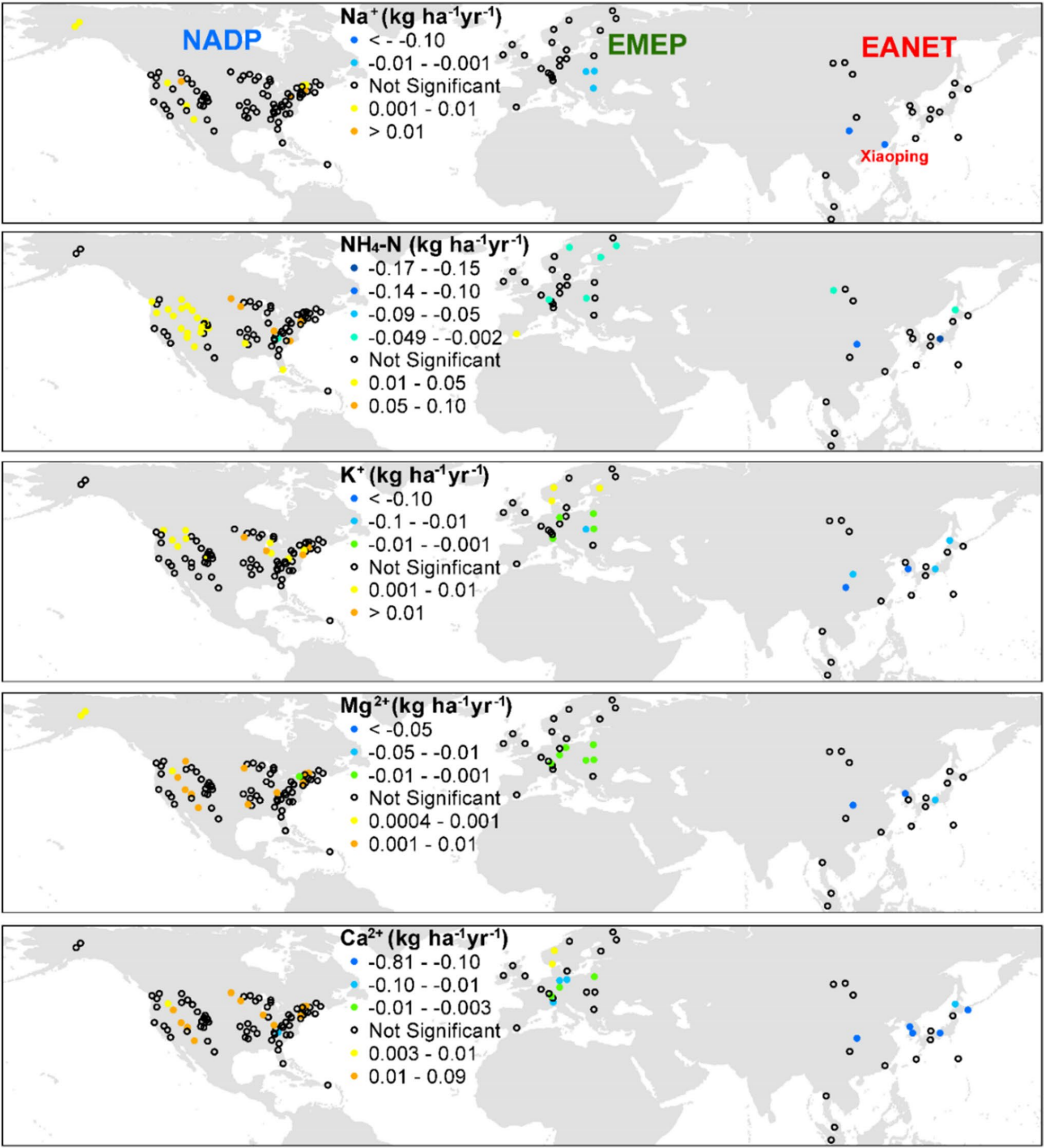
The annual trends of Na^+^, NH_4_-N, K^+^, Mg^2+^, and Ca^2+^ depositions (kg ha^−1^ year^−1^) from top to bottom panels. The color symbols indicate that the trends are statistically significant determined by Mann–Kendall (MK) test at *P* value < 0.05. The values are derived from slope (annual change rate) of linear regression models

Over the past two decades, there is less than one-third of forest sites showing significant increasing of cation depositions in NADP, and only four and five sites exhibited a declining trend in EMEP and EANET which all displayed a dispersal pattern across three networks. Only 15 of the 128 forest sites have significant trends of annual Na^+^ deposition, with the significant changes mainly reflecting changes in precipitation quantity. For example, Xiaoping (site 12) in southeast China showed declines of precipitation, Na^+^ concentration, and deposition (Fig. 3; Fig. S2; Chang et al. 2022). For NH_4_-N depositions, 28 of the 86 (or 33%) of the forest sites exhibit significant increasing trends in NADP and three-fourths of them (21 sites) scattered at western USA (Fig. 3). Midwest USA was the major food production region where livestock manure and nitrogen fertilizer application were not strictly regulated, which likely contributed to the increased ammonium deposition over the past decade (Du et al. 2014). Wildfires could be the equally important source of ammonium. Koplitz et al. (2021) estimated that wildland fire burning emissions contributed 0.2 kg N ha^−1^ year^−1^ on average across the USA during 2008–2012, with maxima 1.4 kg N ha^−1^ year^−1^ in the Northwest, reaching 30% of annual deposition in some regions of Northwest.

**Fig. 4:**
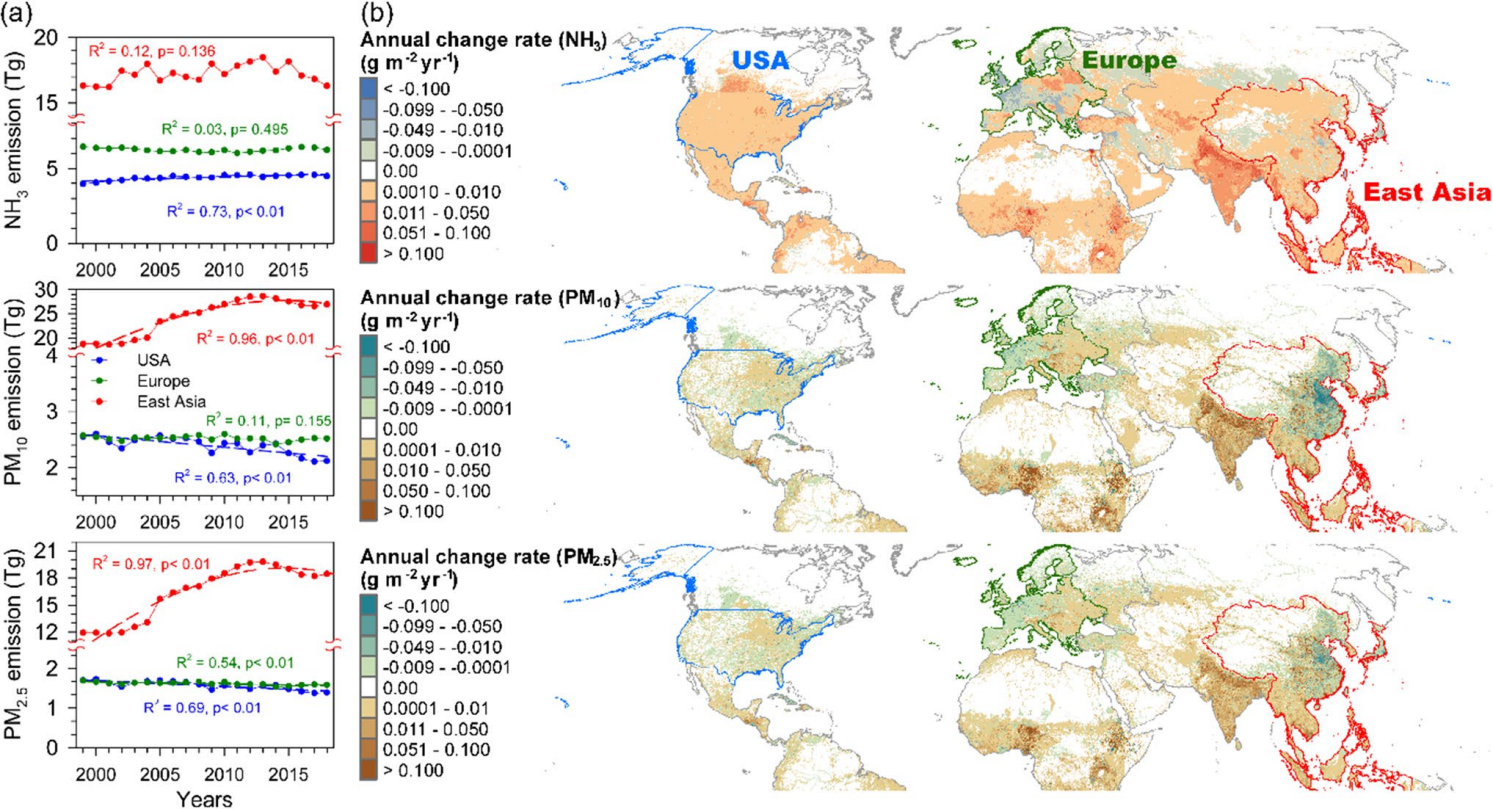
The annual trends of NH_3_, PM_10_, and PM_2.5_ emissions (Tg) (**a**) and their reduction rate (g m^−2^ year^−^.^1^) in the USA, Europe, and East Asia during 1999 and 2018 (**b**) (data source: EDGAR v6.1: https://edgar.jrc.ec.europa.eu/dataset_ap61)

**Fig. 5:**
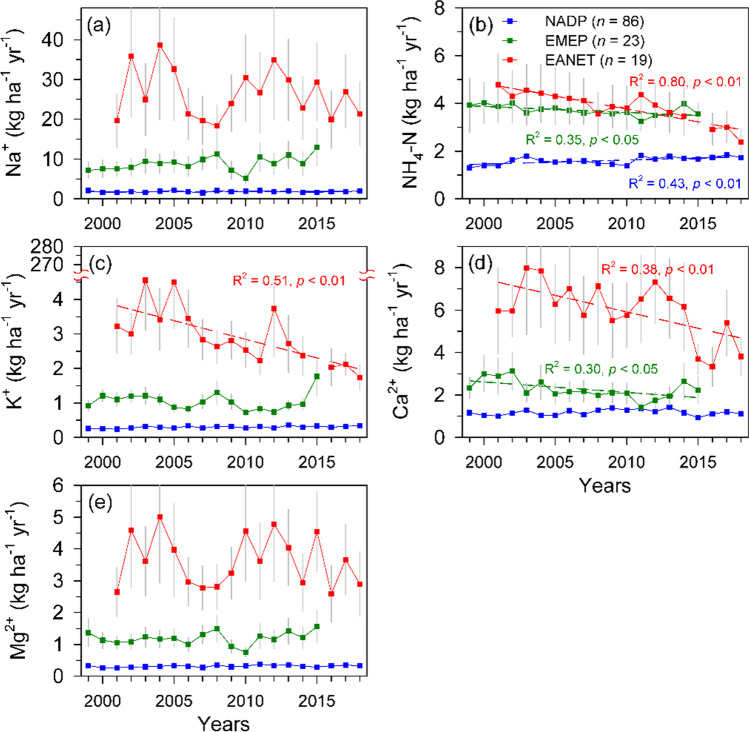
The temporal trends of average of annual depositions of Na^+^ (**a**), NH_4_-N (**b**), K^+^ (**c**), Ca^2+^ (**d**), and Mg^2+^ (**e**) across forest sites of the NADP (*n* = 86), EMEP (*n* = 23), and EANET (*n* = 19) networks during 1999–2018. Gray bars indicate stand errors. The data of NH_4_-N and K^+^ in 2015 of EANET are excluded due to the severe fires occurred in that year

Furthermore, studies indicated that NH_4_-N accounted for more than 60% of total DIN deposition over the conterminous USA owing to the increasing NH_3_ emissions and decreasing NOx emissions and NO_3_-N depositions (Fig. 4; Xing et al. 2013; Li et al. 2016b). The opposite patterns of elevating ammonium deposition and declining nitrate deposition in USA had shifted the DIN deposition from nitrate-dominated to ammonium-dominated over the last decade (Li et al. 2016b; Chang et al. 2022). In contrast, only five and four sites show significant decreasing trends of NH_4_-N deposition in EMEP and EANET, respectively (Figs. 3 and 5). The stably high NH_4_-N depositions have retained the ammonium-dominated DIN deposition across EMEP and EANET (Figs. 2, 3, and 5). Most of EMEP sites show no significant NH_4_-N reduction which is in agreement with the stable NH_3_ emission in Europe (Figs. 3 and 4). In EANET, there is no relationship between NH_4_-N deposition and NH_3_ emissions (Figs. 3, 4, and 5), which might be due to high variation of NH_3_ emissions across the region. The decreases of SO_2_ and NOx emissions after 2013 in Asia and China had been documented (Ma et al. 2019), which might contribute to stabilization or reduction of NH_4_-N deposition due to the close relationship between acid ions and ammonium (Mkadam et al. 2008; Kopáček et al. 2016; Chang et al. 2022). However, the total emissions of SO_2_ and NO_x_ in East Asia remain 3–4 times of the levels in the USA and Europe, and current total NH_3_ emission in East Asia is still 2–3 times higher than those in USA and Europe (Fig. 4).

For the other three BCs, K^+^, Mg^2+^, and Ca^2+^, approximately 20% (25/128, Mg^2+^ and Ca^2+^) to 23% (30/128, K^+^) of the forest sites exhibit significant trends of annual depositions over last two decades (Fig. 3). The forest sites with increasing trends of cations most scattered in NADP (14–17 sites), whereas the sites with decreasing trends mainly distributed in central and east Europe and locations near urbans in EANET (Fig. 3). Studies have demonstrated that dust emissions from industrial activities were an important source of BCs, and their depositions usually decreased with the reductions of pollutant emissions (Duan et al. 2013; Kopáček et al. 2016). The declining in the deposition of the three cations in central and east European forest sites and forest sites near urban in Asia is parallel with the declining deposition of sulfate and nitrate in a previous analysis, which might postpone the recovery of precipitation acidity (Chang et al. 2022). Although studies suggested that the particulate matter had contribution of base cations of approximately 30–60% (Werner et al. 2014; Edgerton et al. 2020). Yet the significant decreases of PM_10_ and PM_2.5_ emissions in USA and Europe are not parallel with the insignificant trends of the three cations across NADP and EMEP (Figs. 3, 4, and 5), possibly because the current cationic depositions have been reaching a relatively low stable level compared to EANET, and as well as the emissions (Figs. 2, 4, and 5). The high spatial variations, reduction in east and coastal China and increase in west China and Southeast Asia, might explain the inconsistency because China is a developing country with an incomplete construction of infrastructures such as unpaved roads, soil exposure, or over-farming in remote regions particularly (Zhang et al. 2020). Another possible reason is that all forest sites are selected by natural background at landscape scale, and most forest sites of NADP and EMEP scattered in remote locations far from anthropogenic sources. However, there are fewer monitoring sites across EANET, especially in China, and two sites in central China (Jiwozi and Jinyunshan) are not only close to hotspot of air pollutant emissions and human activities but also influenced by dust transport from northwest China, which significantly contributed to ammonium and cation depositions (Figs. 1 and 2; Duan et al. 2016; Du et al. 2018; Zhang et al. 2020; Chang et al. 2022).

The large emission reduction strategies such as end-of-pipe treatment measures, shutting down small factories, and strengthening of environmental supervision of several 5-year plans in China resulted in reductions of both acidic pollutants and BCs (Liu and Wang 2017). But the current total depositions of BCs in EANET are 3 to 6 times higher than the levels in NADP and EMEP which may reflect the much higher total emissions of NH_3_ and particulate matter in East Asia than in the USA and Europe (Figs. 2, 4, and 5b). In regions with low chemical weathering rate of rock such as North America, North Europe, and Northeast China, high cationic depositions are an important source of acidifying buffering capacity (Duan et al. 2002, 2016). If the cationic depositions decline more rapidly than acid depositions, a further deterioration of soil and ecosystems would be expected (Larssen and Carmichael 2000). Several long-term studies in Europe and North America have illustrated that the recovery of stream acidification shows a delay for many decades from acidic deposition (Keller et al. 2019; Patel et al. 2020; Rosseland 2021; Webster et al. 2021; Ahrends et al. 2022). Therefore, the trends of natural and anthropogenic cation emission deserve more attention following S and N reductions in East Asia.

### The spatiotemporal patterns of neutralizing factor (NF)

**Fig. 6:**
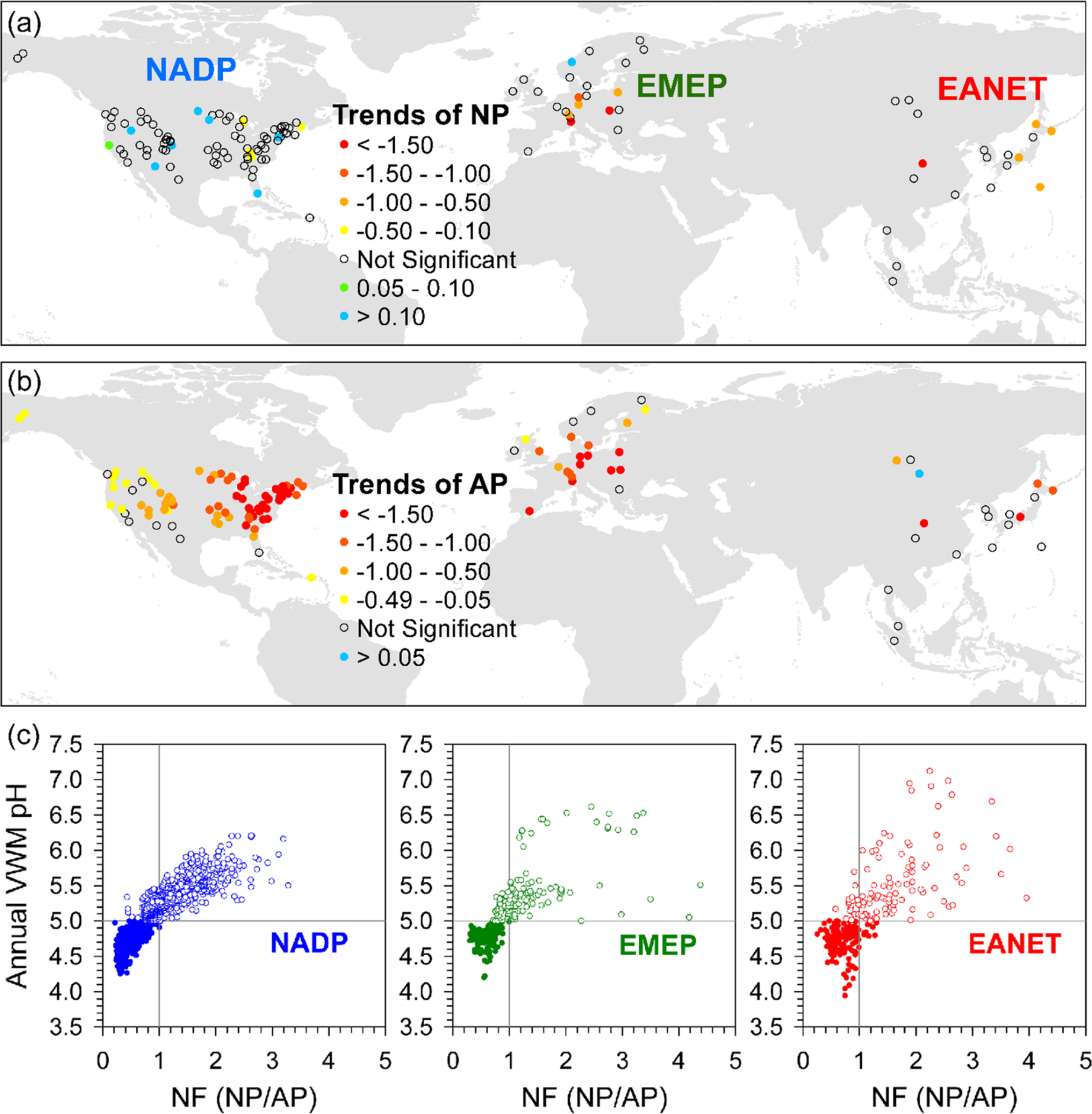
The annual trends of NP (**a**) and AP (**b**) and the distributions of annual VWM pH against NF (NP/AP) for each site among NDAP (*n* = 86), EMEP (*n* = 23), and EANET (*n* = 19) from left to right panels over the past two decades (**c**). The color symbols indicate that the trends are statistically significant determined by Mann–Kendall (MK) test at *P* < 0.05 in panels (**a**) and (**b**), and their values are derived from slope (annual change rate) of linear regression models. The NP sums up concentrations (μeq L^−1^) of nssCa^2+^, nssMg^2+^, nssK^+^, and NH_4_-N, and the AP includes concentrations of nssSO_4_-S and NO_3_-N in panel (**c**), and open and filled circles stand for annual VWM precipitation pH > 5.0 and < 5.0, respectively. Please refer to Fig. 1 for the locations

**Fig. 7:**
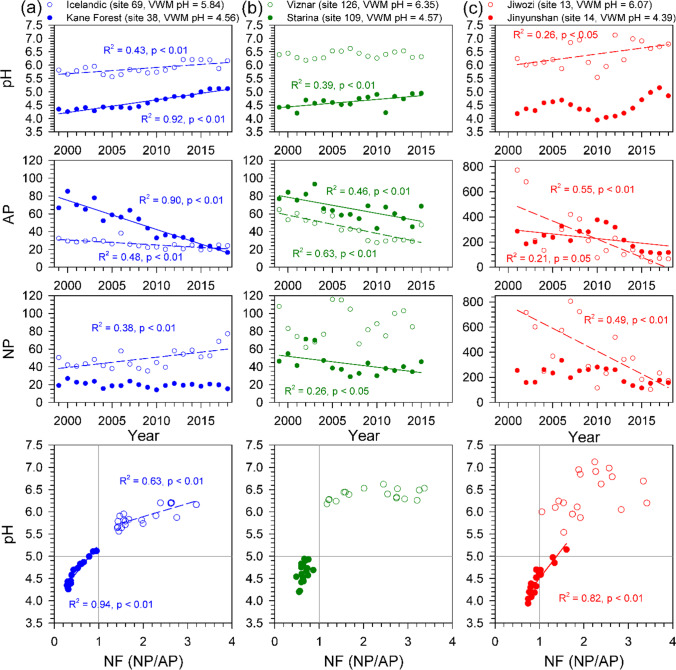
The highest and lowest VWM precipitation pH sites selected from NADP (**a**; Icelandic and Kane Forest), EMEP (**b**; Viznar and Starina), and EANET (**c**; Jiwozi and Jinyunshan) networks, and their annual trends of annual precipitation pH, concentration of acid potential (AP, [nssSO_4_-S + NO_3_-N]), major neutralization potential (NP, [nssCa^2+^  + nssMg^2+^  + nssK^+^  + NH_4_-N]), and NF (NP/AP) against pH from top to bottom panels. Open and filled circles stand for the sites of highest and lowest VWM precipitation pH, respectively. Dash and solid lines indicate the significant regression lines at *P* < 0.05 for the sites with the highest and lowest VWM precipitation pH, respectively. Please refer to Fig. 1 for their locations

During the study period, most of the sites show divergent patterns between NF and precipitation pH. When the NF is larger than 1.0, the annual precipitation VWM pH is mostly > 5.0, whereas when the NF is smaller than 1.0, the annual precipitation VWM pH is mostly < 5.0 across three networks (Fig. 6). We further selected two sites with the highest and lowest VWM precipitation pH from each monitoring network to illustrate the trends of AP, NP, NF, and precipitation pH over the study period. The sites are Icelandic and Kane Forest (NADP), Vizar and Starina (EMEP), and Jiwozi and Jinyunshan (EANET) (Fig. 7). The AP varied greatly from 40–800 μeq L^−1^ (Jiwozi) to < 30 μeq L^−1^ (Icelandic), and NP also varied largely from 60–700 μeq L^−1^ (Jiwozi) to about 20 (Kane Forest) (Fig. 7). For the highest VWM pH of three sites (5.54–6.35), there were similar patterns of declining AP accompanying with increasing or higher NP which results in NF larger than unity, and consequent precipitation pH > 5.5 (Fig. 7). For the lowest VWM pH (4.39–4.57) of three sites, their APs also show a significant declining trend. However, the much lower or more rapid decreasing of NPs lead the NFs to < 1.0 in most years in which precipitation pH was below 5.0 (criterion of acid rain; Galloway et al. 1982). Studies suggested that nssCa^2+^, nssMg^2+^, nssK^+^, and NH_4_-N are all important in buffering acidity of precipitation (Rodhe et al. 2002; Mkadam et al. 2008; Duan et al. 2016; Keresztesi et al. 2019). The region with high acid depositions might be neutralized by high cation deposition that led to neutral precipitation acidity such as the sites in EANET, whereas the locations with low cation depositions would be more sensitive to variations of subtle acid depositions such as in forests in NADP and EMEP (Fig. 7).

**Fig. 8:**
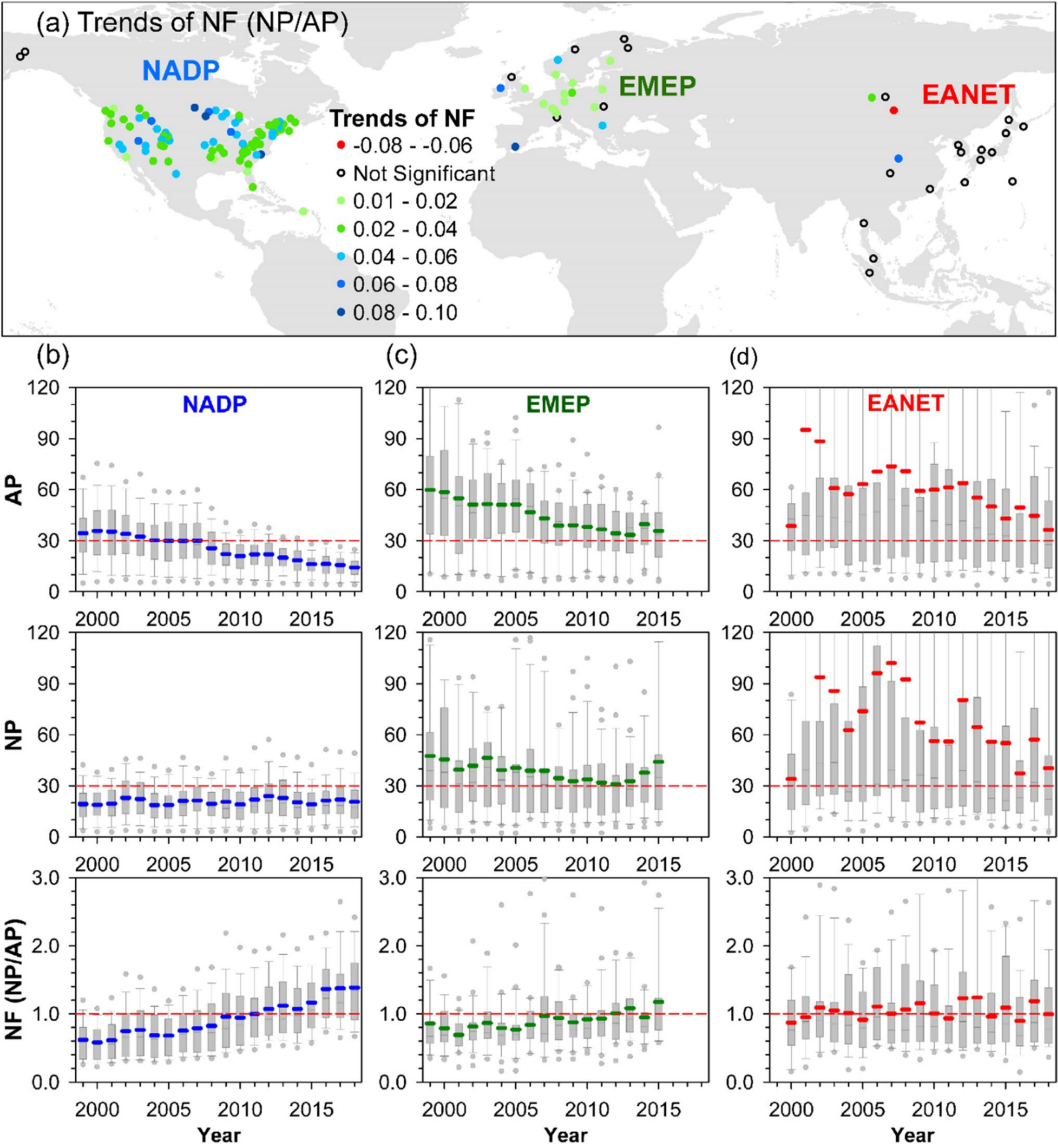
The patterns of annual trends of neutralization factor (NF) (**a**), acid potential (AP, [nssSO_4_-S + NO_3_-N]), major neutralization potential (NP, [nssCa^2+^  + nssMg^2+^  + nssK^+^  + NH_4_-N]), and NF (NP/AP) across three monitoring networks of NADP (**b**), EMEP (**c**), and EANET (**d**). Filled solid circles indicate significant temporal trends based on Mann–Kendall (MK) test at *P* < 0.05. The bold blue, green, and red lines in panels (**b**)–(**d**) represent mean value of each annual boxplot

For NF, 103 of the 128 forest sites exhibit an increasing trend, 84 in NADP, 17 in EMEP, and 2 in EANET, while only one (Tereji in EANET) shows a decreasing trend (Fig. 8). The increasing NF is a widespread phenomenon in the conterminous USA. For example, many sites in northeast (west) America with average NF < 0.3 (~ 1.1) in the first 5 years have elevated to close 1.0 (> 1.5) in recent years (Fig. 8b). The increasing NF trends in NADP are mainly caused by the declines in AP (sulfate and nitrate; Fig. 8a and b). Although 17 sites in EMEP show increasing NF, the concurrent reduction of NP leads to limited improvement of NF over time, except locations at southern Europe (Fig. 8). In contrast, the high variability of AP and NP leads to the lack of a clear pattern of NF at most locations in EANET which constrains the recovery of buffering capacity, with two-thirds of the sites having precipitation pH lower than 5.0 (Chang et al. 2022).

In addition to locations near arid regions such as deserts where the dust transportation is the main source of BC, anthropogenic emissions have a large contribution to BC depositions in urban and developed areas (Vet et al. 2014; Johansen et al. 2019). The increases of NFs in NADP reflect the rapid reduction of sulfate and nitrate and some elevation of nutrient cations compared to EMEP and EANET, attributable to their long-run emission control policies (Figs. 3, 6, and 8; Chang et al. 2022). A study conducted in central Europe indicated that the dust emission control measures had been initiated by regulating major industrial and energy sources since early 1980s (Kopáček et al. 2016). However, the SO_2_ and NOx emission regulation started in the post-communist countries in 1990s that delayed the increase of precipitation pH, which was different from Western Europe (Kopáček et al. 2016). After the late of 1990s, the further concurrent decreases of AP and NP result in some improvement but stagnant NF (Fig. 8), and the precipitation pH of half sites remained low (Chang et al. 2022). From the observations in EANET, both natural and anthropogenic factors are important to BC deposition. For example, the fluctuations of fires associated with El Niño drought in Indonesia contribute huge amount of emissions of particle matter and consequent NH_4_-N and K^+^ depositions (Fig. 2). In China, the implementation of SO_2_ and NOx emission reduction simultaneously had led to a considerable cutback in BC supply (Wen et al. 2020; Zhang et al. 2020). However, the current highest nssCa^2+^ deposition in central China (Jiwozi and Jinyunshan; Fig. 2) is attributable to the dust transportation from semi-arid regions and industrial emission (Duan et al. 2016; Du et al. 2018). Although recovery of degraded vegetation in semi-arid regions reduced the dust production via wind erosion (Zhang et al. 2016; Du et al. 2018), the recent extreme droughts after 2005 provide large quantities of dust in northern China and supply of BC to offshore and distant downwind regions (Song et al. 2016). Thus, both climate change and land cover change will contribute to the variation of BC emission and deposition.

Since 1990s, East Asia has become the global hotspot of SO_2_, NOx, and BC emissions and vulnerable regions of surface water and soil acidification due to sensitive soils (Rodhe et al. 2002; Monks et al. 2009; Vet et al. 2014; Duan et al. 2016). Regionally, the soil acidifications have been commonly observed in China, Japan, Korea, and Taiwan due to acid deposition, over application of N fertilizers, or low acid buffering capacity of bedrock (Horng and Chang 1996; Yagasaki et al. 2001; Nakahara et al. 2010; Yang et al. 2012). Despite long-term high emissions and depositions of S and N occurred in East Asia, widespread declines of trees and forests have not been observed over the past two decades (Takahashi et al. 2020). Only a few streams of forested watersheds have shown signs of impacts of acidification such as defoliation and stream water acidification in central Japan (Matsubara et al. 2009; Nakahara et al. 2010). To curb S and N emissions is the foremost task to mitigate further acidification in susceptible soil, surface water, and forest ecosystems. Concurrent reductions of BC depositions accompanying with regulations on S and N emission control also need attention as BC deposition is important in replenishing the depletion of BCs from forest ecosystems (Fernandez et al. 2003; Hynicka et al. 2016; Kopáček et al. 2016; Du et al. 2018).

## Conclusions

This study synthesizes the temporal changes and spatial patterns of cation depositions (Na^+^, NH_4_-N, K^+^, Mg^2+^, and Ca^2+^) and their neutralization potential over the last two decades based on 128 forest sites of three monitoring networks, i.e., NADP (*n* = 86), EMEP (*n* = 23), and EANET (*n* = 19). The results showed that the depositions of cationic nutrients were much higher in EANET compared to NADP and EMEP. The sea salt-associated sodium deposition exhibited a significant divergence from marine (> 15 kg ha^−1^ year^−1^) to inland (< 3.0 kg ha^−1^ year^−1^) forest sites attributable to the precipitation and influences of sea spray. The higher NH_3_ and particulate matter emissions in Southeast Asia might have a large contribution to the higher cation depositions in EANET than NADP and EMEP. The annual trends of cations revealed that less than one-third of the forest sites revealed significant trends which the spatial patterns were dispersal across the three networks. Possibly, base cation (BC) depositions have reached a low-stable condition in NADP and EMEP, while it has high spatial variation in the temporal change in EANET. The difference in BC deposition among the three networks reflects their different development of economy. Our analysis finds that the annual trends of neutralization factor (NF) in NADP result from the declining of acidic potential (AP) instead of neutralization potential (NP) as BC deposition has been stably low over the past 20 years. Whereas the concurrent decrements of AP and NP in EMEP or high level of both AP and NP in EANET have come to a standstill of acid neutralizing capacity. The BC depositions play an important role involving the precipitation acidity and the support of nutrient cation for forest ecosystem across regions, and the evolution of BC depositions over time should be taken into consideration of modeling precipitation chemistry and forest health with changing acid deposition.

## Supplementary Information

Below is the link to the electronic supplementary material.

Supplementary file1 (DOCX 2706 KB)

## Data Availability

The sources of all data analyzed in this study are included in this published article and supplementary information files.
