# Supplementary material for: Wet depositions of cations in forests across NADP, EMEP, and EANET monitoring networks over the last two decades
Source: Environ Sci Pollut Res Int. 2022 Nov 12;30(10):26791–806. doi: 10.1007/s11356-022-24129-8 (PMC9995420; doi:10.1007/s11356-022-24129-8)
Supplement: Supplementary file 1 — Supplementary file1 (DOCX 2706 KB) [file 11356_2022_24129_MOESM1_ESM.docx]

**Supplementary materials**

Wet depositions of cations in forests across NADP, EMEP and EANET monitoring networks over last two decades

Chung-Te Chang^1,2^, Ci-Jian Yang^3^, Jr-Chuan Huang^4^

^1^ Center for Ecology and Environment, Tunghai University, Taichung 40799, Taiwan

^2^ Department of Life Science, Tunghai University, Taichung 40799, Taiwan

^3^ German Research Centre for Geosciences (GFZ), Potsdam 14473, Germany

^4^ Department of Geography, National Taiwan University, Taipei 10617, Taiwan

Corresponding author.

E-mail addresses: chungtechang@thu.edu.tw (Chung-Te Chang).


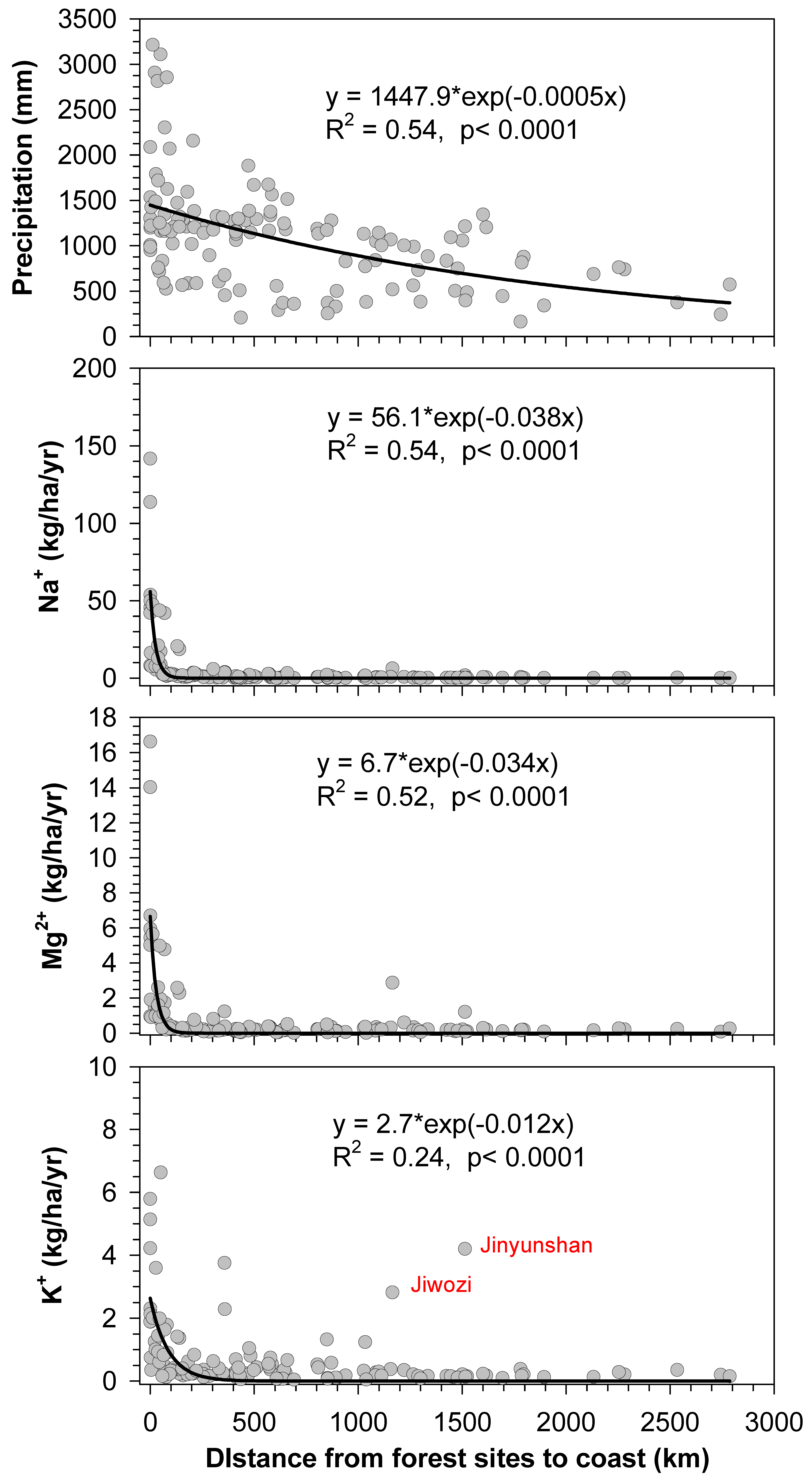


**Fig. S1** The average annual precipitation (mm), average annual fluxes (kg/ha/yr) of sea-salt associated depositions (Na^+^, Mg^2+^, and K^+^) against the distance from forest sites to coast (km). The best regression lines are based on exponential equations


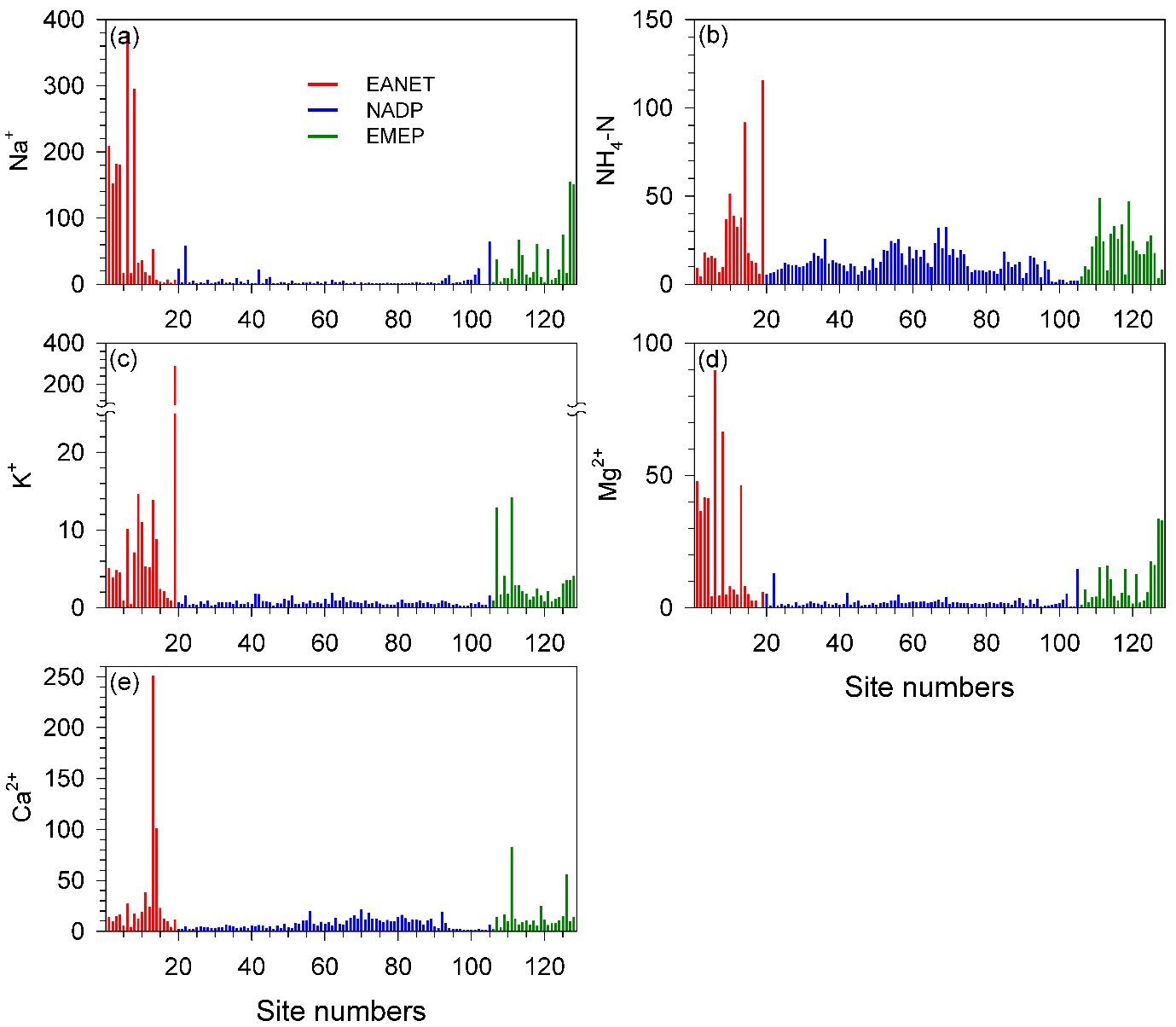


**Fig. S2** The original precipitation ionic concentrations (μeq L^‑1^) of forest sites across EANET (2001-2018), NADP (1999-2018), and EMEP (1999-2015). Please refer to Fig. 1 for the number of sites
